# The antiSMASH database version 4: additional genomes and BGCs, new sequence-based searches and more

**DOI:** 10.1093/nar/gkad984

**Published:** 2023-10-30

**Authors:** Kai Blin, Simon Shaw, Marnix H Medema, Tilmann Weber

**Affiliations:** The Novo Nordisk Foundation Center for Biosustainability, Technical University of Denmark, Lyngby 2800, Denmark; The Novo Nordisk Foundation Center for Biosustainability, Technical University of Denmark, Lyngby 2800, Denmark; Bioinformatics Group, Wageningen University, Wageningen, 6708PB, The Netherlands; The Novo Nordisk Foundation Center for Biosustainability, Technical University of Denmark, Lyngby 2800, Denmark

## Abstract

Many microorganisms produce natural products that are frequently used in the development of medicines and crop protection agents. Genome mining has evolved into a prominent method to access this potential. antiSMASH is the most popular tool for this task. Here we present version 4 of the antiSMASH database, providing biosynthetic gene clusters detected by antiSMASH 7.1 in publicly available, dereplicated, high-quality microbial genomes via an interactive graphical user interface. In version 4, the database contains 231 534 high quality BGC regions from 592 archaeal, 35 726 bacterial and 236 fungal genomes and is available at https://antismash-db.secondarymetabolites.org/.

## Graphical Abstract

**Graphical Abstract:**
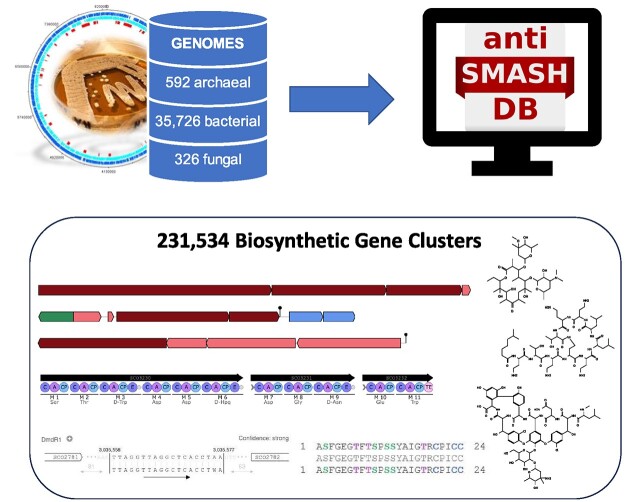

## Introduction

Secondary metabolites produced by microorganisms are the main source of bioactive compounds that are in use as antimicrobial and anticancer drugs (1), as well as fungicides, herbicides, pesticides and other crop protection agents (2). Classically, these compounds were discovered by making extracts out of samples from natural sources, followed by chemical isolation, purification and activity screening. The sequencing boom of the last decade has made microbial genome data readily available, making it possible to complement this traditional approach with genome mining technologies (3). Software tools for natural product genome mining have existed for over a decade (as discussed in various reviews (4–8)). Only a few databases made such data available, starting with the now-defunct ClusterMine360 (9) in 2013.

Since its initial release in 2011, antiSMASH (10–16) has become the most widely used tool for genome mining for secondary/specialised metabolites and is generally regarded as the gold standard. antiSMASH uses a rule-based approach to detect genome regions containing biosynthetic gene clusters based on conserved biosynthetic enzymes from (currently) 88 different biosynthetic pathway types. In addition to cluster-specific analyses for many of the better-understood pathways, antiSMASH also compares identified regions to the MIBiG database (17) of known BGCs, as well as a dataset of antiSMASH results predicted from publicly available genomes.

antiSMASH is a genome mining tool by design, meaning that it analyses and annotates individual microbial genomes, one at a time. To help with research questions that can better be answered with cross-genome datasets, we developed the antiSMASH database (18–20). With its easy to use query builder, it allows researchers to quickly run even complex queries against BGCs identified across tens of thousands of genomes. Additionally the database is used as the basis for antiSMASH’s ClusterBlast functionality, with ClusterBlast hits linking to the database. antiSMASH results are cross-referenced to similar results in the database, as well as to similar clusters from the MIBiG database.

Here we present the fourth version of this database, covering 592 archaeal, 35 726 bacterial and 236 fungal genomes.

## Materials and methods

### Selection of included genomes

Archaeal, bacterial and fungal genomes were downloaded from the NCBI RefSeq database on 4–5 April 2023, using the ncbi-genome-download tool (21) using the ‘complete’, ‘chromosome’ and ‘scaffold’ assembly levels. To avoid issues with badly fragmented assemblies negatively affecting BGC prediction quality, assemblies with >100 (for archaea and bacteria)/150 (fungi) contigs were discarded. Redundancy filtering was performed as described previously (20), with the exception that Mash (22) was now used for all taxa, including fungi. After redundancy filtering, 641 archaeal, 38 991 bacterial and 257 fungal sequences remained.

### antiSMASH annotations and data import

On this filtered dataset, we used GNU parallel (23) to run antiSMASH 7 with the options ‘$\hbox{-\,-}$cb-knownclusters $\hbox{-\,-}$cb-subclusters $\hbox{-\,-}$cc-mibig $\hbox{-\,-}$clusterhmmer $\hbox{-\,-}$tigrfam $\hbox{-\,-}$pfam2go $\hbox{-\,-}$rre $\hbox{-\,-}$asf $\hbox{-\,-}$tfbs’. antiSMASH successfully processed 640 archaeal, 38 940 bacterial and 255 fungal assemblies, the others were skipped due to the lack of gene annotations or other annotation-related errors. From this first run, we extracted all predicted ribosomally synthesised and posttranslationally modified peptide (RiPP) precursors and regions to build new CompaRiPPson and ClusterBlast datasets, respectively. After creating the updated datasets, antiSMASH 7 was re-run on the first round's results using the options ‘$\hbox{-\,-}$cb-general $\hbox{-\,-}$reuse’, also updating CompaRiPPson results with the new dataset.

The SQL schema for the database (https://github.com/antismash/db-schema/) and importer (https://github.com/antismash/db-import/) were updated to support antiSMASH 7 results. During the import process, assemblies without any antiSMASH predictions were dropped, resulting in the final count of 592 archaeal, 35 726 bacterial and 236 fungal assemblies being represented in the database.

## Results and discussion

The NCBI RefSeq database contains a wealth of microbial genomes. However, the database does contain a lot of redundancies caused by tens of thousands of sequences of common pathogens like *Escherichia coli*, *Salmonella enterica* or *Staphylococcus aureus*. Additionally, many of the genome assemblies are draft assemblies from short reads that leave the genome in hundreds or even thousands of tiny contigs, which has massive impacts on the quality of BGC detection (24). In order to have a good representation of BGCs across the whole sequenced microbial tree of life without overly biassing for the frequently sequenced species, and to ensure that clusters are as complete as possible without being spread over many contigs, the antiSMASH database applies rigorous quality filtering and sequence-similarity based filtering. After filtering and processing, the fourth version of the antiSMASH database contains 231 534 high-quality BGCs from 592 archaeal, 35 726 bacterial and 236 fungal high-quality, representative, genomes. Annotations were performed using antiSMASH 7.1, which has additional rules on top of those in the 7.0 release (16): isocyanides, NRP-related isocyanides, highly-reducing PKS type IIs, darobactins, triceptides, archaeal RiPPs and hydrogen cyanides. This results in a total of 88 different supported pathways.

Version 4 of the database makes all of the antiSMASH predictions available using its query functionality. The NRPS/PKS module search has been integrated into the regular query builder to allow for combined queries like ‘find regions containing NRPS modules with *N*-methyltransferase domains in the genus *Streptomyces*’ or any other filters on top of the module selection. During user testing of the simple text query, we noticed confusion about which search categories were covered, as well as frustration about the absence of type completion hints. As the current version of the database brings the number of supported search categories up to 39, we decided to remove the simple text query. This means that all database queries now run via the query builder interface.

**Figure 1.**
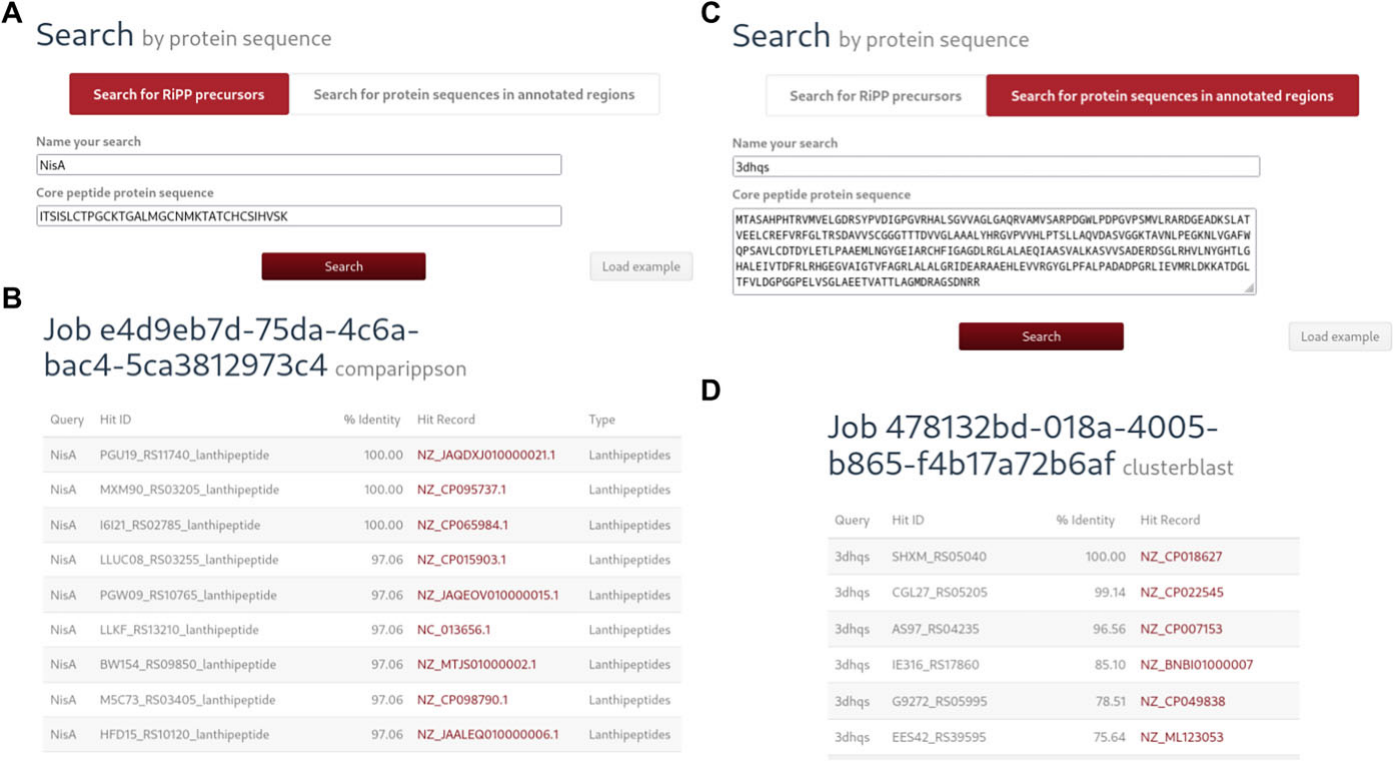
(**A**) The RiPP precursor search loaded with the lanthipeptide nisin A’s core sequence. (**B**) The CompaRiPPson results for the above search (**C**) The protein sequence search using a 3-dehydroquinate synthase. (**D**) The protein sequence search results

For users who want to search the database using their own protein sequence data or known RiPP precursor sequences, two newly added search features are available. The RiPP precursor search (Figure 1A) uses NCBI blast+ blastp (25) to compare a user-provided sequence with all predicted RiPP precursors in the database (Figure 1B). Similarly, the protein sequence search (Figure 1C) uses DIAMOND (26) to compare user-provided sequences to all protein sequences from predicted BGCs (Figure 1D).

The development work needed to allow running these kinds of searches in the background has also been used to make CSV and FASTA downloads more reliable. Previously, queries returning CSV or FASTA results were generally slow and needed strict pagination to return data before the browser connection timed out. This in turn made these queries cumbersome to use, especially from automated scripts. In version 4 of the database, we instead create background processes to collect all of the requested data. Once collected, data is available from the antiSMASH database servers for a week before being cleaned up automatically. This should make it drastically easier to download larger slices of the antiSMASH database.

Additionally, the complete dataset for the whole database is available for download on our download server in various formats for users wishing to integrate any or all of the data into their own in-house tools, see the data availability statement for details. Examples on how to use the antiSMASH database search and download functions can be found on the database's ‘Help’ page.

Compared to version 3′s 25 802 assemblies, version 4 contains 36 554, roughly a 42% increase. At the same time, the number of high-quality BGCs increased, increasing the number of BGCs that did not run into a contig edge from 147 517 to 231 534, almost 57%. This increase is likely due to the increased number of BGC types supported by the latest version of antiSMASH.

## Conclusions

Genome mining continues to be an invaluable technique for assessing microbial biosynthetic potential. Since 2011, antiSMASH has aided with these efforts. The antiSMASH database helps to compare identified clusters across genomes and allows for more complex searches to contextualise and cross-reference findings via a user-friendly web interface.

With a selection 231 534 BGC regions from archaea, bacteria and fungi, the antiSMASH database version 4 is a comprehensive collection of secondary/specialised metabolite biosynthetic gene clusters with up-to-date, high quality antiSMASH-based annotations available to the natural product research community.

## Data Availability

The antiSMASH database is available at https://antismash-db.secondarymetabolites.org/. There are no access restrictions for academic or commercial use of the web server. The source code components and SQL schema for the antiSMASH database are available on GitHub (https://github.com/antismash) under an OSI-approved Open Source license. The complete set of antiSMASH results, the antiSMASH JSON files, and an SQL dump of the database can be downloaded from the antiSMASH download server (https://dl.secondarymetabolites.org/database/4.0/).
